# G-Quadruplex Structures Formed by Expanded Hexanucleotide Repeat RNA and DNA from the Neurodegenerative Disease-Linked *C9orf72* Gene Efficiently Sequester and Activate Heme

**DOI:** 10.1371/journal.pone.0106449

**Published:** 2014-09-10

**Authors:** Jason C. Grigg, Nisreen Shumayrikh, Dipankar Sen

**Affiliations:** 1 Department of Molecular Biology & Biochemistry, Simon Fraser University, Burnaby, British Columbia, Canada; 2 Department of Chemistry, Simon Fraser University, Burnaby, British Columbia, Canada; Department of Pathology, Anatomy & Cell Biology, Thomas Jefferson University, United States of America

## Abstract

The expansion of a (G_4_C_2_)_n_ repeat within the human *C9orf72* gene has been causally linked to a number of neurodegenerative diseases, most notably familial amyotrophic lateral sclerosis (ALS) and frontotemporal dementia (FTD). Recent studies have shown that the repeat expansion alters gene function in four ways, disrupting the gene's normal cellular roles and introducing toxic gain of function at the level of both DNA and RNA. (G_4_C_2_)_n_ DNA, as well as the RNA transcribed from it, are found to fold into four-stranded G-quadruplex structures. It has been shown that the toxicity of the RNA G-quadruplexes, often localized in intracellular RNA foci, lies in their ability to sequester many important RNA binding proteins. Herein we propose that a distinct toxic property of such RNA and DNA G-quadruplexes from the *C9orf72* gene may arise from their ability to bind and oxidatively activate cellular heme. We show that G-quadruplexes formed by both (G_4_C_2_)_4_ RNA and DNA not only complex tightly with heme but also enhance its intrinsic peroxidase and oxidase propensities. By contrast, the antisense (C_4_G_2_)_4_ RNA and DNA neither bind heme nor influence its oxidative activity. Curiously, the ability of *C9orf72* DNA and transcripts to bind and activate heme mirror similar properties that have been reported for the Aβ peptide and its oligomers in Alzheimer's disease neurons. It is therefore conceivable that *C9orf72* RNA G-quadruplex tangles play roles in sequestering intracellular heme and promoting oxidative damage in ALS and FTD analogous to those proposed for Aβ peptide and its tangles in Alzheimer's Disease. Given that neurodegenerative diseases in general are characterized by mitochondrial and respiratory malfunctions, the role of *C9orf72* DNA and RNA in heme sequestration as well as its inappropriate activation in ALS and FTD neurons may warrant examination.

## Introduction

Amyotrophic lateral sclerosis (ALS or Lou Gehrig's Disease) and frontotemporal dementia (FTD) are both serious and significant neurological diseases that appear to have familial forms as well as arising sporadically within populations [1]. Recently, an abnormal expansion of a repeating GGGGCC sequence in the DNA of the *C9orf72* gene was identified in patients with the familial forms of these diseases, but also in a proportion of patients with the sporadic diseases [1]–[3]. This repeat expansion has also been reported in the brains of certain patients of depressive pseudodementia [4], Huntington disease [5], hippocampal sclerosis dementia [6], and non-fluent aphasia [7]. A number of studies have provided insight into how this repeat expansion, at the level of both RNA and DNA, may contribute to ALS and FTD. A circular dichroism and NMR study by Fratta *et al.* [8] showed that the ‘minimal’ repeat sequence [r-(G_4_C_2_)_3_G_4_C, or “C9Gru”] from *C9orf72* RNA forms an intramolecular, parallel-stranded, G-quadruplex fold in the presence of K^+^ ions. A subsequent study by Reddy *et al.* [9] showed that longer repeats of r(G_4_C_2_)_4_ could also form irregular intermolecular multimers, which were proposed to correspond to intranuclear RNA foci observed in ALS neurons. Most recently, a broad-ranging study by Haeusler *et al.* [10] defined a conceptual framework in which multiple roles for this hexanucleotide repeat expansion in disease were invoked. Working with DNA and RNA oligomers corresponding to various pieces of the repeat Haeusler *et al.* [10] showed that *both* the DNA and RNA form highly stable G-quadruplex folds [11]–[13]. G-quadruplexes are four-stranded structures that can form, under physiological conditions, in G-rich DNAs and RNAs (see below). It was proposed that both gain-of-function and loss-of-function toxicity could be linked to repeat expansion within *C9orf72*, at the level of gene (DNA) as well as transcript (RNA). Loss of function at the DNA level could be manifested in poor transcription of *C9orf72*, owing to formation of G-quadruplexes in the gene itself. Loss of function at the RNA level likely occurs from a dearth of the *C9orf72* gene product, owing to inefficient translation of repeat-expanded transcripts folded into stable G-quadruplexes. Gain of function can be contemplated at the level of G-quadruplex-folded *C9orf72* transcripts: (i) sequestering essential RNA-interacting proteins, including splicing factors such as ASF/SF2 and hnRNPA1, and nucleolin; and (ii) from the potential synthesis of toxic dipeptides from the repeat GGGGCC motifs within the transcript [10], [14]. Most notably, Haeusler *et al*. [10] reported a deep proteomic analysis of the cellular proteins sequestered away by the *C9orf72* transcript-containing RNA foci. There were 288 proteins identified in the pull-downs, including nucleolin and heterogeneous nuclear ribonucleoprotein (hnRNP) U, which showed specificity for the G-quadruplex.

G-quadruplexes are a family of highly ordered folded structures formed by single-stranded guanine-rich DNAs and RNAs, of genomic or other origin, under physiological conditions [11]–[13]. Within G-quadruplexes guanines from either a single strand of DNA/RNA or from two, three, or four distinct strands hydrogen bond together *via* Hoogsteen base-pairing to yield guanine base quartets. G-quadruplexes are highly polymorphic, both in terms of strand stoichiometry (forming both inter- and intramolecular structures) and strand orientation/topology. Thus, quadruplexes that are wholly strand-parallel, strand-antiparallel, as well as having other combinations of strand orientation have been reported for DNA. Conversely, RNA G-quadruplexes invariably take on a parallel strand orientation. The K^+^ cation (and, less so, the Na^+^ cation) specifically supports G-quadruplex formation and stability. Other cations present in the cell, such as Mg^2+^ and Ca^2+^, do not specifically support formation of G-quadruplexes but do stabilize them *via* general electrostatic stabilization [15], [16].

G-quadruplexes provide excellent binding surfaces for a variety of large- and small-molecule ligands [17], [18]. Our lab first showed that the ubiquitous cellular cofactor, ferric heme [Fe(III)-protoporphyrin IX], binds tightly to some but not all G-quadruplexes [19], [20], with dissociation constant (K_d_) values as low as ∼10 nM [21]. The most remarkable property of such G-quadruplex•heme complexes (invariably containing parallel or partially parallel-stranded quadruplexes), however, is that the DNA/RNA activates the bound heme for enhanced oxidative activity- frequently to the levels of heme-utilizing proteinaceous enzymes such as peroxidases, peroxygenases, and monooxygenases [20]–[22]. In the presence of low concentrations of oxidizing agents such as hydrogen peroxide, or molecular oxygen aided by cellular reducing agents such as NADH [23] or ascorbate, G-quadruplex•heme complexes catalyze robust one-electron (peroxidase) as well as two-electron (peroxygenase and monooxygenase) oxidation reactions [24].

ALS and neurodegenerative diseases in general are characterized, among other things, by (a) respiratory/mitochondrial dysfunction, and (b) general oxidative stress. It has recently been reported that the Aβ peptide, thought to be the causal agent of Alzheimer's disease, both binds and activates heme towards oxidative activity [25], [26]. These remarkable observations have led to proposals that sequestration of heme by Aβ peptide tangles may, on one hand, constitute a “loss of function” for cellular respiratory/mitochondrial activity, but also an oxidative “gain of function” by way of Aβ-bound heme activation [26]. Given that in *C9orf72*-impacted diseases both the DNA and RNA corresponding to the hexanucleotide repeat expansion fold into G-quadruplexes, we investigated whether such G-quadruplexes also bound heme, concomitantly activating the bound heme towards accelerated oxidative activity.

## Materials and Methods

### Materials

All DNA and RNA oligonucleotides were purchased from University Core DNA Services (University of Calgary), purified with standard desalting methods, and used without further purification. Sequences are listed in Table 1. Oligos were dissolved in 10 mM Tris-EDTA buffer (10 mM Tris pH 7.5, 0.1 mM ethylenediaminetetraactetate (EDTA)) and frozen at -20°C until needed. Hemin was purchased from Frontier Scientific (Logan, UT, USA). Amplex red was purchased from (Santa Cruz Biotechnology Inc., Dallas, TX, USA). All other chemicals were purchased from Sigma-Aldrich.

**Table 1:** Oligonucleotide sequences used in this study.^a^

Name	Sequence
r(G_4_C_2_)_4_	5′ – GGGGCCGGGGCCGGGGCCGGGGC – 3′
r(C_4_G_2_)_4_	5′ – GGCCCCGGCCCCGGCCCCGGCCCC – 3′
d(G_4_C_2_)_4_	5′ – GGGGCCGGGGCCGGGGCCGGGGC – 3′
d(C_4_G_2_)_4_	5′ – GGCCCCGGCCCCGGCCCCGGCCCC – 3′
CatG4	5′ – TGGGTAGGGCGGGTTGGGAAA – 3′

a RNA sequences have an “r” prefix and DNA samples have a “d” prefix.

### Circular dichroism spectroscopy

25 µM DNA and RNA solutions were prepared in 25 mM Tris, pH 7.5. Samples were heated at 95°C for ∼ 5 minutes, removed from heat and allowed to cool to room temperature. CD spectra were obtained using a Jasco J-810 CD spectrometer with a 1 mm pathlength cuvette at 18°C. Scans were recorded over the 200–320 nm wavelength range, and averaged from three scans recorded at a scan rate of 100 nm/min with a 1 nm bandwidth. Potassium chloride was added directly from a stock solution to the sample in question. The sample was then incubated at 18°C until equilibrium had been reached and no further change in the spectrum was observed (∼10 minutes).

### UV-Vis heme binding

UV/Vis spectra were obtained using a Cary 100 UV/Vis spectrophotometer. Hemin stock solutions were freshly prepared at 10 mM in 0.1 M potassium hydroxide. Heme was diluted to 0.5 µM in 40 mM HEPES, pH 8.0, 20 mM potassium chloride, 1% dimethyl formamide, 0.05% Triton X-100. Quadruplex DNA or RNA (25 µM) was prepared in the same buffer by heating at 95°C for 5 minutes and cooling to room temperature. Heme solutions were titrated with RNA and DNA (0–20 µM) and allowed to incubate for at least one hour at 20°C before recording spectra. Dissociation constants (K_d_) were determined using the following equation as described by Wang *et al*. [27]: [DNA]_0_  = K_d_ (A – A_0_)/(A_∞_ – A)+[P_0_] (A – A_0_)/(A_∞_ – A_0_).

### ABTS peroxidation

Quadruplex solutions were prepared as described for the heme binding experiments, in two buffers: 40 mM HEPES, pH 8.0, 20 mM potassium chloride, 1% *N*,*N*-dimethylformamide, 0.05% Triton X-100 (NH_4_-HEPES buffer); and, 25 mM Tris-Cl, pH 8.0, 20 mM potassium chloride, 1% *N*,*N*-dimethylformamide (DMF), 0.05% Triton X-100 (Tris buffer). Peroxidase reactions were set up with 10 µM DNA/RNA G-quadruplex, 0.1 µM heme, 1 mM 2,2′-azino-bis(3-ethylbenzothiazoline-6-sulphonic acid) (ABTS) and 0-5 mM hydrogen peroxide. The reaction was initiated by addition of varying amounts of peroxide and monitored by following absorbance at 413 nm.

### Oxidase activity

Quadruplex solutions were prepared as described for the heme binding experiments, in 40 mM HEPES, pH 8.0, 20 mM potassium chloride, 1% DMF, 0.05% Triton X-100. Reactions were performed in the same buffer supplemented with 10 µM DNA/RNA G-quadruplex and 1 µM heme. Amplex red was added to 0.1 mM, and the solutions were incubated at room temperature for 30 minutes. Reductants [1 mM reduced β-nicotinomide adenine dinucleotide (NADH), 1 mM ascorbate, or 0.1 mM hydrogen peroxide] were then added to the sample and incubated in the dark, at room temperature. Samples were photographed at intervals from 0–24 hrs. To verify that the color changes observed were a result of resorufin production, final absorption spectra were recorded on a Cary 100 UV/Vis spectrophotometer.

## Results

### (G_4_C_2_)_4_ but not (C_4_G_2_)_4_ DNA and RNA fold into G-quadruplexes in the presence of K^+^ ions

**Figure 1:**
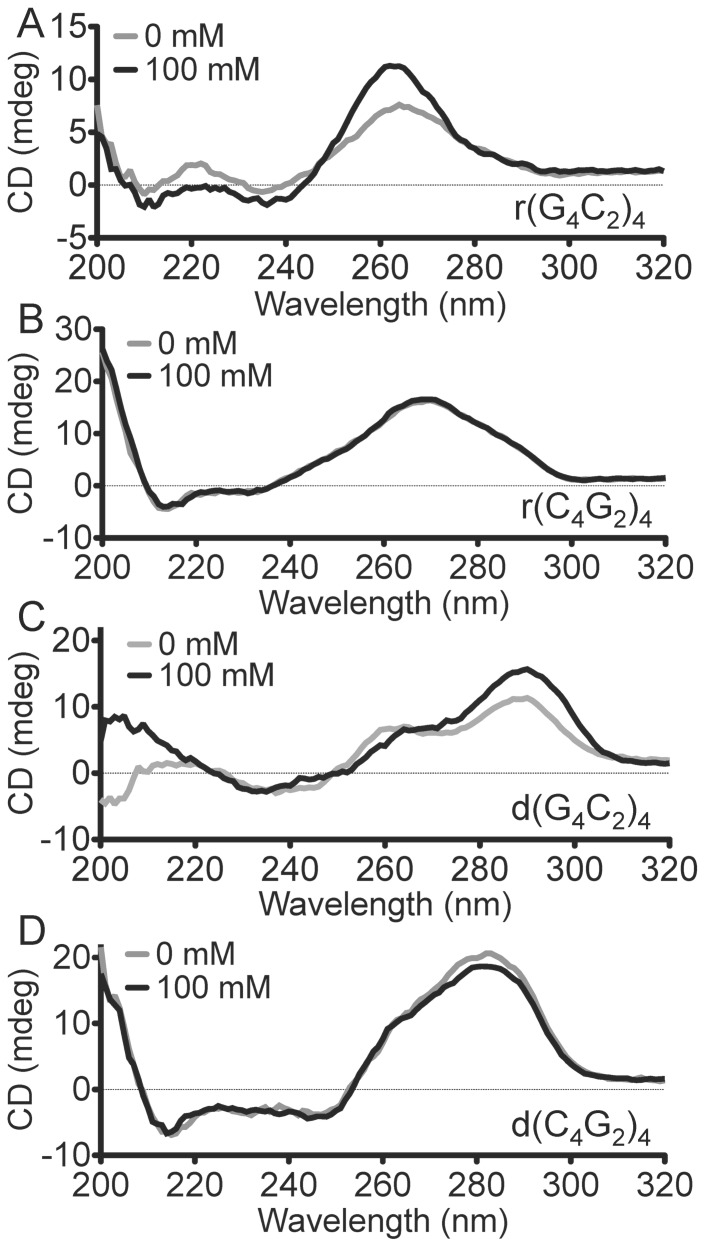
G-repeat expansion RNA and DNA form G-quadruplexes in the presence of potassium. UV Circular Dichroism spectra of (**A**) r(G_4_C_2_)_4_, (**B**) r(C_4_G_2_)_4_, **(C**) d(G_4_C_2_)_4_, and (**D**) d(C_4_G_2_)_4_ in 25 mM Tris, pH 7.5, in the presence of either 0 mM or 100 mM KCl.

Circular dichroism was used to investigate secondary structure formation by the four oligonucleotides, d(G_4_C_2_)_4_, r(G_4_C_2_)_4_, d(C_4_G_2_)_4_, and r(C_4_G_2_)_4_. Figure 1 shows the data. The CD spectrum of each oligonucleotide, at 25 µM concentration, was examined in solution in 25 mM Tris, pH 7.5 (grey lines, Figure 1), as well as in 25 mM Tris, pH 7.5, 100 mM KCl (black lines, Figure 1). Panels B and D show that both r(C_4_G_2_)_4_ and d(C_4_G_2_)_4_ give CD spectra that do not change upon the addition of potassium, suggesting their likely single-stranded/Watson-Crick duplex composite structures are unchanged with or without KCl. Panels A and C, however, show that both r(G_4_C_2_)_4_, and d(G_4_C_2_)_4_ show characteristic features of G-quadruplex formation. Thus, r(G_4_C_2_)_4_ shows an enhanced positive peak at 260 nm and a negative peak at ∼ 240 nm. These are consistent with its forming a parallel-stranded quadruplex, consistent with earlier reports (8–10), and also with the requirement that RNA G-quadruplexes be parallel-stranded. The DNA oligomer, d(C_4_G_2_)_4_, shows a major positive peak at 295 nm and a lesser one at 260 nm, as well as a negative peak at ∼235 nm. Such a spectrum is consistent with the formation of a conformer mixture of G-quadrupexes of both antiparallel (positive peak at 295 nm) and parallel (positive peak at 260 nm) strand orientation. The formation of such a conformer mixture is consistent with the known polymorphism of DNA G-quadruplexes, whereby the formation of a given fold is acutely sensitive to DNA sequence, concentration, as well as to salt identity and concentration. In a recent study Haeusler *et al*. [10] reported that d(G_4_C_2_)_4_, at a much lower DNA concentration (4 µM) than the 25 µM used in this study, formed primarily an antiparallel quadruplex; however, they also found that the CD spectra of oligomers in the series d(G_4_C_2_)_n_, where n  =  3, 6, or 10, gave K^+^-generated parallel/antiparallel conformer mixtures with composite CD spectra similar to that shown in Figure 1, panel A. Our data and those of Haeusler *et al*. [10], are therefore not mutually inconsistent.

### G-quadruplexes formed by d(G_4_C_2_)_4_ and r(G_4_C_2_)_4_ bind heme

We carried out UV-vis spectroscopy experiments to investigate whether in the presence of K^+^ the four oligonucleotides, d(G_4_C_2_)_4_, r(G_4_C_2_)_4_, d(C_4_G_2_)_4_, and r(C_4_G_2_)_4_, bound heme. We, and others, had previously reported that many G-quadruplexes, particularly those with parallel or mixed parallel/antiparallel strand orientations, complexed strongly with heme [21], [28], [29]. The salient spectroscopic characteristics of heme binding by G-quadruplexes (in experiments in which a fixed heme concentration is titrated with increasing concentrations of G-quadruplex) are (a) a large hyperchromicity as well as red-shift (from ∼398 nm to 402–404 nm) of the dominant Soret absorption peak of the heme, and (b) characteristic changes in the heme visible spectra. Cumulatively, these features of G-quadruplex•heme complexes strongly resemble the spectroscopic features of the bound heme within natural hemoenzymes such as metmyoglobin and horseradish peroxidase (HRP) [20].

**Figure 2:**
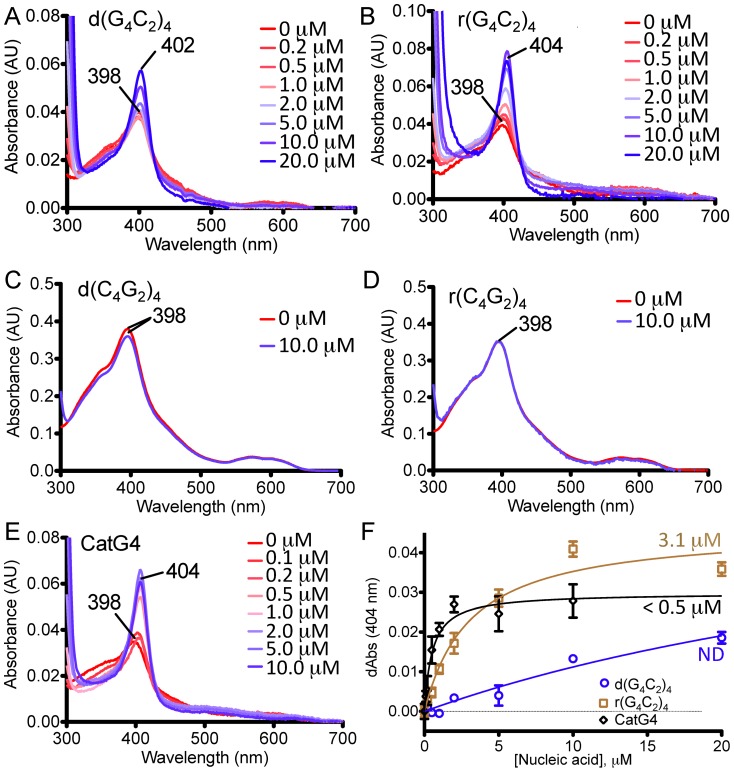
G-repeat expansion RNA and DNA bind heme. UV-visible spectroscopy of fixed concentrations of heme (0.5 µM) titrated and equilibrated with progressively increasing concentrations of DNA/RNA. (**A**) d(G_4_C_2_)_4_, (**B**) r(G_4_C_2_)_4_, (**C**) d(C_4_G_2_)_4_, (**D**) r(C_4_G_2_)_4_, (**E**) CatG4. Panel **F** shows plots of A_404nm_ from each of the plots shown in **(A)–(E)**, as functions of the DNA/RNA concentration.

Figure 2, panels A–D, show the effect of titrating 0–20 µM of d(G_4_C_2_)_4_, r(G_4_C_2_)_4_, d(C_4_G_2_)_4_, or r(C_4_G_2_)_4_ into a buffered HEPES solution (pH 8.0) with 0.5 µM heme and 20 mM K^+^. A known parallel G-quadruplex forming DNA, CatG4 (5′-TGG GTA GGG CGG GTT GGG AAA-3′) was separately examined as a positive control, under identical conditions (panel E). It is evident that d(G_4_C_2_)_4_, r(G_4_C_2_)_4_, as well as CatG4 (panels A, B, and E) show the Soret peak hyperchromicity and red-shift features that are indicative of heme-binding by these oligonucleotides. The plots in panel F of Figure 2 reveal that the repeat expansion RNA oligonucleotide, r(G_4_C_2_)_4_, binds heme with a dissociation constant, K_d_, of ∼3 µM. d(G_4_C_2_)_4_ also binds heme, albeit with a weaker affinity than its RNA counterpart. By comparison, the known parallel-stranded DNA G-quadruplex, CatG4, binds heme strongly (K_d_<0.5 µM). In contrast to the above three G-rich oligonucleotides the two cytosine-rich oligonucleotides, d(C_4_G_2_)_4_ and r(C_4_G_2_)_4_, show none of the characteristic spectroscopic features of heme binding.

### Complexes of heme with d(G_4_C_2_)_4_ and r(G_4_C_2_)_4_ show enhanced peroxidase activity

**Figure 3:**
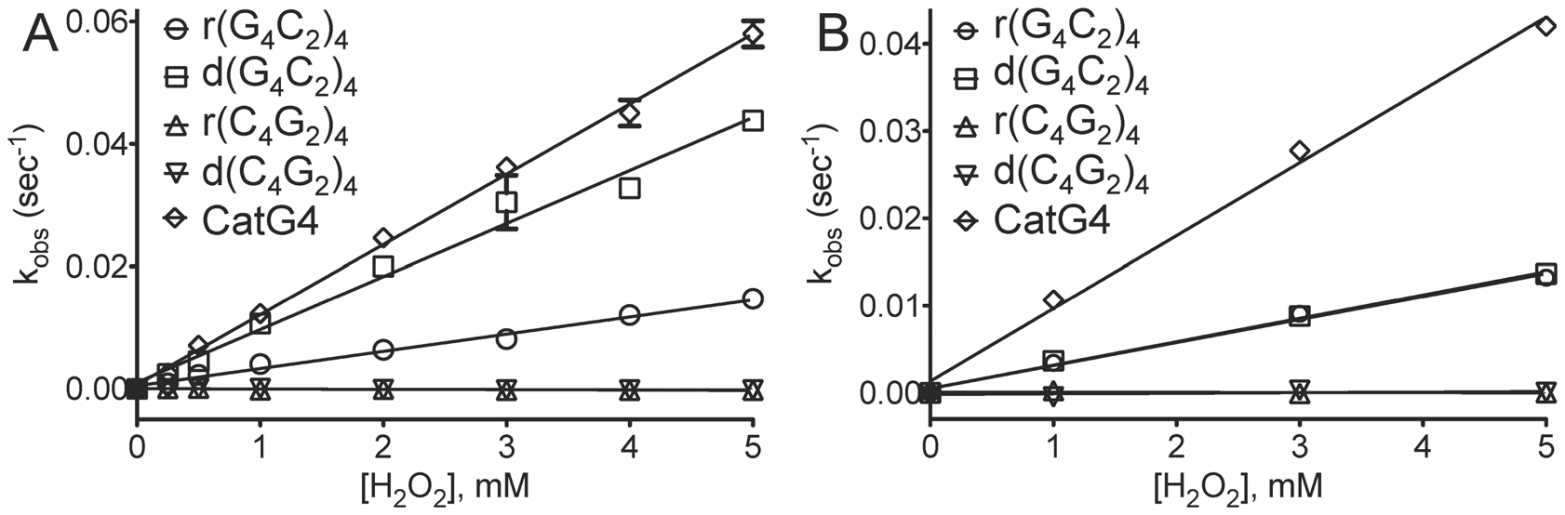
*c9orf72* repeat DNA And RNA catalyze peroxidase reactions. k_obs_ values for peroxidation reactions made up of 10 µM DNA/RNA, 0.1 µM heme, 1 mM ABTS and varied hydrogen peroxide concentrations from 0-5 mM. Panel **A** reactions were carried out in NH_4_-HEPES buffer (40 mM HEPES, pH 8.0, 20 mM potassium chloride, 1% *N*,*N*-dimethylformamide, 0.05% Triton X-100); and, Panel **B** reactions were carried out in Tris buffer (25 mM Tris-Cl, pH 8.0, 20 mM potassium chloride, 1% *N*,*N*-dimethylformamide, 0.05% Triton X-100).

Our original discovery that some G-quadruplexes complexed strongly with heme [19], [20] came with an unexpected corollary: such complexes showed a 10^2^–10^3^-fold, G-quadruplex-enhanced, peroxidase (one-electron oxidation) activity over the low intrinsic peroxidase activity of monomeric heme. In the presence of micromolar to millimolar concentrations of hydrogen peroxide and a chromogenic reducing substrate, such as 2,2′-azino-bis(3-ethylbenzothiazoline-6-sulphonic acid) (ABTS), heme•G-quadruplex complexes show vigorous oxidation of the colorless ABTS to generate the green ABTS^•+^ radical cation species. This specifically G-quadruplex-enhanced peroxidase activity of the bound heme requires only a buffered solution containing K^+^ ions; however optimal activity is enabled by the presence, in addition to K^+^, of NH_4_^+^ [20] or intracellular amines such as putrescine, spermidine or, especially, spermine [30]. Figure 3 plots the measured peroxidase activities of heme (reported as k_obs_ values), in the presence of the four *C9orf72*-related oligomers, as functions of hydrogen peroxide concentration. Figure 3, panel A, shows peroxidation rates in the optimal buffer (10 µM DNA/RNA and 0.1 µM heme in 40 mM NH_4_-HEPES, pH 8.0, 20 mM potassium chloride, 1% dimethyl formamide, 0.05% Triton X-100). Under these conditions, it can be seen that heme in the two C-rich oligomer [r(C_4_G_2_)_4_ and d(C_4_G_2_)_4_] solutions show no detectable peroxidase activity. Heme complexed with the two G-rich oligomers, r(G_4_C_2_)_4_ and d(G_4_C_2_)_4_, however, both show substantial peroxidase activity– the DNA•heme complex greater than the RNA•heme complex.

Figure 3, panel B, shows the peroxidation activities of the above DNA/RNA•heme complexes in the potassium-only buffer solution (25 mM Tris-Cl, pH 8.0, 20 mM KCl, 1% dimethyl formamide, 0.05% Triton X-100). Here, too (though the overall peroxidation rate constants are lower than in the optimal buffer) the same qualitative patterns are observed- the C-rich oligomers do not activate the heme towards peroxidase activity, whereas the G-rich oligomers do. Interestingly, in the Tris/potassium buffer the G-rich oligomers, r(G_4_C_2_)_4_ and d(G_4_C_2_)_4_, activate their complexed heme moiety to approximately the same degree.

### d(G_4_C_2_)_4_•heme and r(G_4_C_2_)_4_•heme complexes also display enhanced oxidase activity

**Figure 4:**
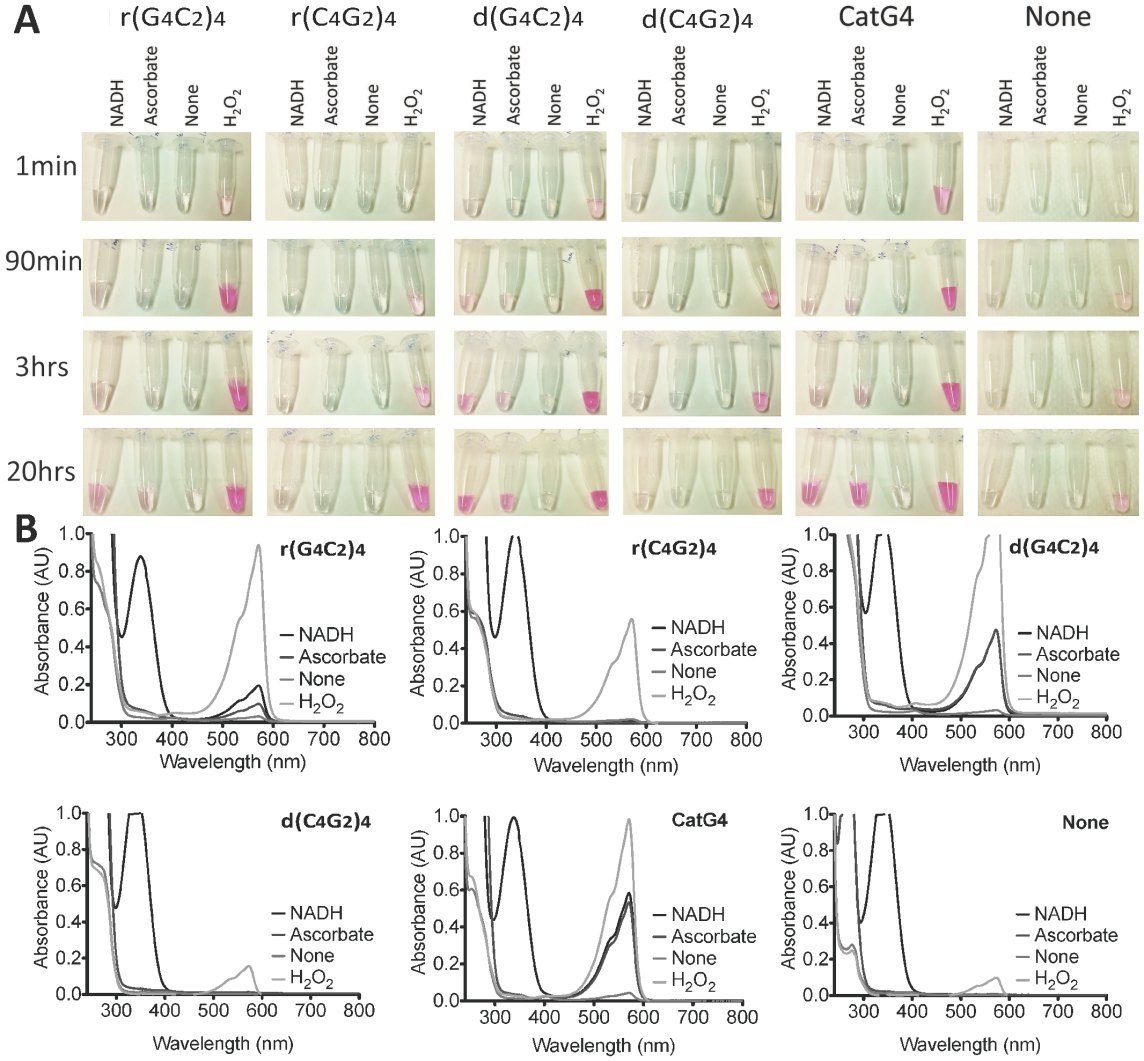
*c9orf72* repeat DNA and RNA catalyze oxidase reactions with NADH and ascorbate. (**A**) A photographic record of the oxidase activity of different DNA/RNA solutions in the presence of heme. Amplex Red oxidation to resorufin produces an intense red color. Each solution containing DNA/RNA (10 µM) and heme (1 µM) was incubated with 1 mM Amplex Red in the presence of NADH or Ascorbate (1 mM), the absence of a reductant or hydrogen peroxide (0.1 mM). (**B**) UV/Vis spectra for samples from panel **A** at ∼24 hrs, showing characteristic spectra for resorufin (l_max_ ∼570 nm).

The above peroxidase activity displayed by the heme complexes of r(G_4_C_2_)_4_ and d(G_4_C_2_)_4_ depends on the availability of hydrogen peroxide. However, some G-quadruplexes complexed with heme have also been reported to display an oxidase activity, whereby they can harness ambient dioxygen (O_2_) gas in the presence of the cellular reductant, nicotine adenine dinucleotide (NADH) [23]. We investigated whether the *C9orf72* repeat expansion-linked DNA and RNA sequences were capable of utilizing heme and ambient oxygen to manifest an oxidase activity. We also investigated whether a different cellular reductant, ascorbate, could take the place of NADH in this reaction. A particularly sensitive means for monitoring oxidase reactions is *via* the oxidative deacetylation of a fluorogenic substrate, Amplex Red, to a bright red product, resorufin. Figure 4, panel A, shows time-lapse photographs of reactions containing Amplex Red, 10 µM oligonucleotide and 1 µM heme in NH_4_-HEPES buffer containing potassium chloride (*vide infra*) supplemented with 1 mM of ascorbate or NADH, or 0.1 mM hydrogen peroxide as a control. In reactions containing r(G_4_C_2_)_4_ and d(G_4_C_2_)_4_, or the control DNA G-quadruplex, CatG4, colour appears rapidly (< 1 min) in the presence of hydrogen peroxide, and within 90 min-20 hrs in the presence of NADH or ascorbate. No colour is seen in these DNA solutions in the absence of any added reductant (labeled as “None”). In reactions incorporating the C-rich oligonucleotides, r(C_4_G_2_)_4_ and d(C_4_G_2_)_4_, or in a solution where no oligonucleotide is present, no resorufin colour develops in the reactions containing NADH, ascorbate, or no added reductant; and, appears relatively slowly (1–90 min) with hydrogen peroxide. That the observed red colour in all of the above experiments does correspond to resorufin is confirmed in the spectra shown in Figure 4, panel B (the resorufin absorption peaks at ∼ 550 nm).

## Discussion

A major underpinning of the postulated gain of function by the repeat expanded *C9orf72* gene in familial ALS and FTD is the formation of G-quadruplexes by the G-rich transcript. It has been convincingly shown that RNA G-quadruplexes and their aggregates, as found in the intracellular RNA foci, are efficient binders of a variety of cellular proteins, and likely serve to sequester away such proteins from their natural functions within the cell. Here, we hypothesize an additional pair of “gain of function” attributes for such RNA G-quadruplexes in ALS, FTD, as well as the other diseases linked to the repeat expansion of the *C9orf72* gene [10]. In this study, we have shown that G-quadruplexes formed by both the RNA and the single-stranded, “sense” strand of DNA from the *C9orf72* repeat expansion (a) bind heme with sufficient affinity to possibly sequester heme away from key cellular (chiefly, mitochondrial and respiratory) functions; and (b) these RNA- and DNA G-quadruplexes serve to chemically activate the bound heme towards catalyzing oxidative reactions, using either hydrogen peroxide or naturally dissolved oxygen as oxidant. Both the G-quadruplex mediated sequestration and activation of heme reported herein occur under physiologically plausible conditions, in terms of solution components, salt, temperature and pH. Furthermore, the neurons from a broad spectrum of neurodegenerative diseases have been found to be significantly enriched with reactive oxygen species (ROS), including hydrogen peroxide, relative to unaffected neurons [31], [32]. Thus, repeat expansion RNA/DNA-sequestered heme may threaten the local cellular environment of ALS and FTD neurons with enhanced oxidative damage. Indeed, this is the first proposal of a role in human disease for the heme-binding and -activating propensities of G-rich nucleic acids.

It has been estimated that in normally functioning human cells there exist > 300,000 DNA motifs potentially capable of folding to G-quadruplexes [33]. However, whatever proportion of these actually do fold to G-quadruplexes may play a constitutive role in the normal trafficking of, especially, regulatory heme [26] from the mitochondria to other loci within the cell. By contrast, the profuse generation of RNA transcripts from the hexanucleotide-expanded *C9orf72* gene in ALS and FTD neurons, which has been observed to cause the assembly and persistence within the cytosol of aggregated RNA tangles and foci, may supply significantly higher numbers of heme-binding and –activating sites within these cells.

As a rule, iron dysfunction and respiration defects appear to be common features of neurodegenerative diseases. Heme constitutes 95% of functional iron in the human body [34]. Jeong *et al*. [35] have reported that dysregulation of iron homeostasis leads to ALS progression in a mouse model for the disease. Deterioration of mitochondrial function, with concomitantly lowered availability of hemes b, c-c1, and a-a3 have also been reported in an ALS yeast model system by Gunther *et al*. [36]. Regarding oxidative damage, there is a strong body of evidence that oxidative damage may play a role in the pathogenesis of neuronal degeneration in both sporadic and familial ALS. [37]. The frequent association of a defective form of superoxide dismutase (SOD) with ALS itself can lead to high levels of intracellular hydrogen peroxide. Liu *et al*. [38] showed that mice transfected with a defective, ALS-linked human SOD gene had heightened intracellular concentrations of hydrogen peroxide and hydroxide radical relative to superoxide.

Curiously, the above-described binding as well as activation of heme by RNA and DNA G-quadruplexes from the *C9orf72* gene may find a curious parallel with the observed affinity for heme shown by the monomer and aggregates of the Aβ peptide, causative agents of Alzheimer's disease. Work by Atamna and colleagues has demonstrated altered metabolism of heme found in the brains of Alzheimer's Disease patients and heme activation by monomeric Aβ peptide as well as its oligomeric aggregates [25], [26], [39], [40]. Indeed, these authors have claimed a significant role for the sequestration and activation of heme in the overall Alzheimer's disease. Aβ has been found to bind two molecules of heme, with K_D_ values of ∼7 and ∼3 µM, respectively [41]. These numbers are remarkably similar to our own measured K_D_ value of 3.1 µM for the binding of heme to r(G_4_C_2_)_4_. It is therefore conceivable that the Aβ peptide and the *C9orf72*-derived G-quadruplex RNA play equivalent roles in heme sequestration and activation in Alzheimer's Disease and in Familial ALS and FTD, respectively.

How strong is the peroxidase activity of heme•G-quadruplex complexes? A study by Klibanov and colleagues [42] found that while a commonly used (but non-biological) substrate such as ABTS was oxidized notably more slowly by heme•G-quadruplexes than by horseradish peroxidase, for certain substrates such as phenolic compounds (for instance, tyrosine, and by extension tyrosine-containing peptides and proteins) heme•G-quadruplexes were superior oxidizing catalysts relative to horseradish peroxidase. Thus, the oxidative damage potential of putative heme•G-quadruplex complexes within cells is by no means negligible.

In this paper we have explored the properties of primarily monomeric, intramolecular folds of the d(G_4_C_2_)_4_ and r(G_4_C_2_)_4_ oligonucleotides. Longer repeats of these same sequences have been shown to form, additionally, into large, parallel-stranded G-quadruplex aggregates [9], [10]. It has been suggested that the RNA foci observed in the neurons of ALS and FTP patients may correspond structurally to these larger G-quadruplex aggregates [9], [10]. Based on earlier observations we fully expect that the larger, irregular aggregates also bind and activate heme. Recently, G-quadruplex aggregates whose formation has been promoted by spermine, have been shown to display superior peroxidation properties relative to their unaggregated counterparts, besides enjoying enhanced longevity for the active heme moiety within the aggregation milieu [30].

Might it be feasible to prevent or interfere with the heme binding and activation properties of the *C9orf72* RNAs, as well as, potentially, other intracellular toxic RNAs that may participate in disease processes? Pearson and coworkers recently reported the interesting observation that a cationic porphyrin, 5,10,15,20-tetra(*N*-methyl-4-pyridyl) porphyrin (TMPyP4), which is known to be an excellent binder of G-quadruplexes as well as of other DNA and RNA folds, sufficiently altered the structure of r(G_4_C_2_)_n_ quadruplexes to impact on the latters' ability to sequester away cellular proteins [43]. To date, a very large number of G-quadruplex binding small molecule ligands have been reported in the literature [17], [18], [44]. It is possible that the identification of a ligand, or ligands, that interfere with heme binding to RNA G-quadruplexes, may contribute positively to therapeutic strategies aimed at neurodegenerative diseases such as ALS and FTD.
